# The Effects of Sports Drinks During High-Intensity Exercise on the Carbohydrate Oxidation Rate Among Athletes: A Systematic Review and Meta-Analysis

**DOI:** 10.3389/fphys.2020.574172

**Published:** 2020-12-11

**Authors:** Xudong Li, Wanxia Wang, Rui Guo, Anqi Wang, Chaojun Wei

**Affiliations:** ^1^Department of Physical Education, Lanzhou University, Lanzhou, China; ^2^The Institute of Clinical Research and Translational Medicine, Gansu Provincial Hospital, Lanzhou, China

**Keywords:** dietary carbohydrates, lipids, oxidation, energy drinks, athletes, meta-analysis

## Abstract

**Background:** This study examines the effects of sports drinks ingestion during high-intensity exercise for carbohydrate oxidation rate (CHO-O) among athletes.

**Methods:** PubMed, Embase, and the Cochrane library were searched for available papers published up to November 2019. The primary outcome is the carbohydrate oxidation rate (CHO-O), and the secondary outcome is the fat oxidation rate (Fat-O). Statistical heterogeneity among the included studies was evaluated using Cochran's Q test and the I^2^ index. The random-effects model was used for all analyses, regardless of the I^2^ index.

**Results:** Five studies are included, with a total of 58 participants (range, 8–14/study). All five studies are randomized crossover trials. Compared to the control beverages, sports drinks have no impact on the CHO-O of athletes [weighted mean difference (WMD) = 0.29; 95% CI, −0.06 to 0.65, *P* = 0.106; I^2^ = 97.4%, *P* < 0.001] and on the Fat-O of athletes (WMD = −0.074; 95% CI, −0.19 to 0.06, *P* = 0.297; I^2^ = 97.5%, *P* < 0.001). Carbohydrate–electrolyte solutions increase CHO-O (WMD = 0.47; 95% CI, 0.08–0.87, *P* = 0.020; I^2^ = 97.8%, *P* < 0.001) but not Fat-O (WMD = −0.14; 95% CI, −0.31 to 0.03, *P* = 0.103; I^2^ = 98.2%, *P* < 0.001). Caffeine has a borderline effect on Fat-O (WMD = 0.05; 95% CI, 0.00–0.10, *P* = 0.050).

**Conclusions:** Compared with the control beverages, sports drinks show no significant improvement in CHO-O and Fat-O in athletes. Carbohydrate–electrolyte solutions increase CHO-O in athletes but not Fat-O.

## Introduction

Energy drinks, also denominated as sports drinks, generally refer to a class of beverages containing sugar and various combinations of ingredients purported to “energize” the body and mind. Such drinks have become widespread in recreational and elite athletes due to their proposed ergogenic effects. A variety of energy drinks are designed to have optimal levels of carbohydrate (CHO) for glycogen replenishment and electrolytes for ion maintenance and prevention of dehydration. They are currently available on the market and are publicized to increase the energy level of the individuals consuming them (Rahnama et al., 2010). Because sports drinks are well recognized for their effect of delaying fatigue during prolonged exercise, various studies have been performed to investigate their performance-enhancing mechanisms (Ishak et al., 2012; Salinero et al., 2014; Shearer and Graham, 2014; Alsunni, 2015).

A previous study has demonstrated that the rate of carbohydrate absorption could exceed 1.2 g/min during exercise when only glucose is fed (Jeukendrup, 2017). Moreover, by ingesting mixtures of glucose and fructose, exogenous carbohydrate oxidation (CHO-O) rates have been demonstrated to increase 1.2–1.7-fold during prolonged exercise (Jentjens and Jeukendrup, 2005). Those studies indicate the efficacy of carbohydrate ingestion for enhancing performance during exercise. Other studies have also investigated the effect of sports drinks on performance during exercise (Byars et al., 2010; Rahnama et al., 2010; Hornsby, 2011; Brink-Elfegoun et al., 2014; Orru et al., 2018). Elite athletes represent a special population in whom the basal metabolism and energy utilization during exercise are different from that of the general population (Sjodin et al., 1996; Koshimizu et al., 2012; Trexler et al., 2014). In addition, the available studies about sports drinks in athletes report conflicting results.

With the recent public interest in the accuracy of the claims of commercially available sports drinks about their benefits for substrate oxidation among athletes, the present meta-analysis is designed to examine the effects of sports drinks ingestion on CHO-O rate among athletes.

## Methods

### Literature Search

This meta-analysis was conducted according to the Preferred Reporting Items for Systematic Reviews and Meta-Analyses (PRISMA) guidelines. Relevant articles were searched using the PICO principle, followed by screening on the basis of the inclusion and exclusion criteria: (1) population—elite athletes with high-endurance prolonged exercise; (2) interventions—sports drink; (3) control—placebo or no control group; (4) study type—cohort study, randomized control trial, case–control study, and crossover study; and (5) language was limited to English. PubMed, Embase, and the Cochrane library were searched for available papers published up to November 2019 using the MeSH keywords “Energy drinks,” “Athletes,” as well as relevant keywords.

### Data Extraction

Study characteristics (authors, year of publication, the country where the study performed, study design, disease type, and sample size), treatment parameters (beverage type and volume of ingestion), the primary outcome (CHO-O), and secondary outcome [fat oxidation rate (Fat-O)] were extracted from the included studies. The selection and inclusion of studies were performed in two stages by two independent reviewers (Anqi Wang and Wanxia Wang). This included the analysis of titles/abstracts, followed by the full texts. Disagreements were resolved by a third reviewer (Rui Guo).

### Quality of the Evidence

The level of evidence of all articles was assessed independently by two authors (Xudong Li and Chaojun Wei), using the Cochrane tool for assessing the risk of bias, as specified in the Cochrane Handbook (Higgins et al., 2019).

### Data Synthesis

Three out of five included studies poorly reported their statistical parameters, and thus, we decided to estimate the means from the provided figures and standard deviations for means of outcome value by using the coefficient of variance (CV) (Jacobs and Viechtbauer, 2017). After excluding studies with imputed standard deviations for all different outcome parameters, the estimates were reasonably robust for standard deviation imputation.

### Statistical Analysis

All analyses were performed using the STATA SE 14.0 software (StataCorp, College Station, TX, USA). Weighted mean difference (WMD) and corresponding 95% confidence intervals (CIs) were used to compare the outcomes. Statistical heterogeneity among the included studies was evaluated using Cochran's Q test and the I^2^ index. I^2^ > 50% and *P* < 0.10 in the Q test indicated high heterogeneity. The random-effects model was used for all analyses, regardless of the I^2^ index. *P* < 0.05 are considered statistically significant. We planned to assess potential publication bias by funnel plots and Egger's test but actually did not do so because the number of studies included in every meta-analysis is fewer than 10, in which case, the funnel plots and Egger's test could yield misleading results and are not recommended (Higgins and Green, 2011).

## Results

### Study Selection

**Figure 1:**
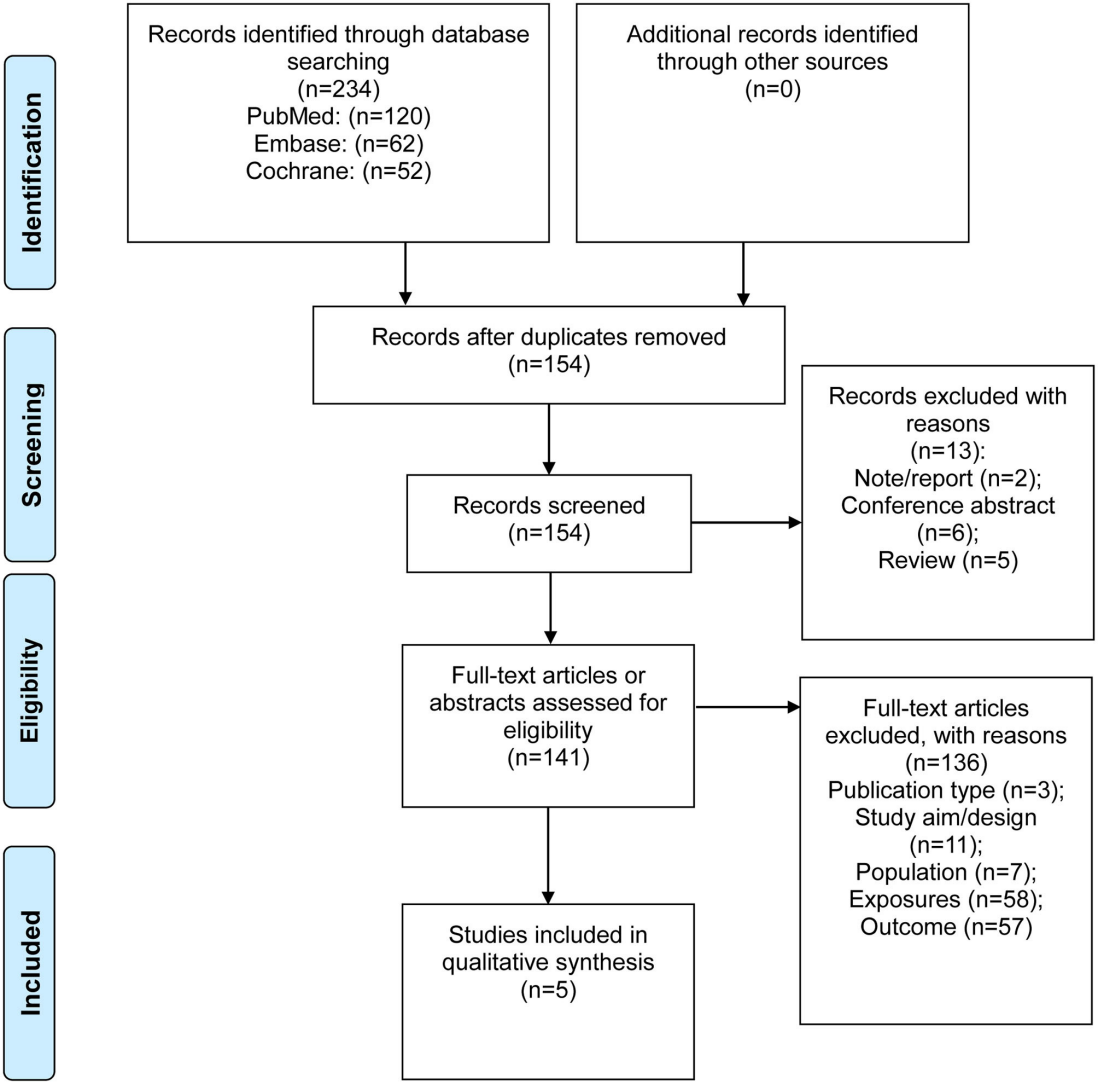
Literature search process.

Figure 1 presents the study selection process. A total of 234 records were retrieved from PubMed, Embase, and the Cochrane Library, and 154 records were left after removing the duplicates. Two records were excluded because they were note/report, six because they were conference abstracts, and five because they were reviews. Then, 141 full-text papers were assessed, and 136 were excluded because of the publication type (*n* = 3), study aim or design (*n* = 11), study population (*n* = 7), exposures (*n* = 58), and outcomes (*n* = 57). Therefore, five studies (Clarke et al., 2005; Hodgson et al., 2013b; Roberts et al., 2014; Garnacho-Castano et al., 2018; Pettersson et al., 2019) are included in the present meta-analysis. Some studies fell short in terms of quality, owing to small numbers of participants, unclear reporting of study methods, and reporting of data in a format that was not easy to combine with other data.

### Characteristics of the Included Studies

Table 1 presents the characteristics of the five studies (Clarke et al., 2005; Hodgson et al., 2013b; Roberts et al., 2014; Garnacho-Castano et al., 2018; Pettersson et al., 2019). There were a total of 58 participants (range, 8–14/study). All five studies are randomized crossover trials. Three studies used a carbohydrate–electrolyte solution (500, 270, and 220 ml), one used caffeine (600 ml), and one used beetroot juice (70 ml). The participants' mean age ranges from 25 ± 3 to 41 ± 7 years. All five studies report the CHO-O and Fat-O. All five trials are from Europe. Supplementary Table 1 presents the Cochrane criteria for the evaluation of randomized controlled trials. All included studies (Clarke et al., 2005; Hodgson et al., 2013b; Roberts et al., 2014; Garnacho-Castano et al., 2018; Pettersson et al., 2019) have a high risk of selection bias regarding the randomization, two studies (Clarke et al., 2005; Pettersson et al., 2019) have a high risk of selection bias regarding allocation concealment, and one study (Hodgson et al., 2013b) has a high risk of performance bias regarding the blinding of the participants and personnel.

**Table 1:** Characteristics of the included studies.

						**Sample size**					
**References**	**Country**	**Study design**	**Type of beverage**	**Consumption per test**	**Sports**	**Total**	**Treatment**	**Control**	**Male**	**Mean age (years)**	**Outcome of interest**
Clarke et al. (2005)	United Kingdom	Randomized crossover	Carbohydrate electrolyte solution	500 ml	Soccer	12	12	12	12	25 ± 3	CHO-O, Fat-O
Hodgson et al. (2013a,b)	United Kingdom	Randomized crossover	Caffeine	600 ml	Cycling	8	8	8	NR	41 ± 7	CHO-O, Fat-O
Roberts et al. (2014)	United Kingdom	Randomized crossover	Carbohydrate–electrolyte solution	270 ml	Cycling	14	14	14	14	31.8 ± 10	CHO-O, Fat-O
Garnacho-Castano et al. (2018)	Spain	Randomized crossover	Beetroot juice	70 ml	Cycling	12	12	12	12	39.3 ± 7.5	CHO-O, Fat-O
Pettersson et al. (2019)	Sweden	Randomized crossover	Carbohydrate electrolyte solution	220 ml	Ski	12	12	12	6	25.2 ± 5	CHO-O, Fat-O

*CHO-O, carbohydrate oxidation rate; Fat-O, fat oxidation rate*.

### Sports Drinks Have No Significant Effect on CHO-O

**Figure 2:**
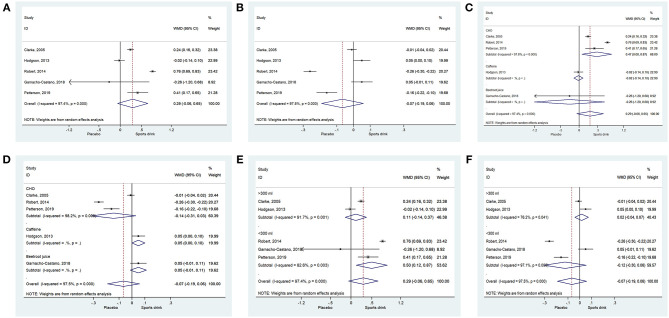
**(A)** Forest plot of CHO oxidation; **(B)** forest plot of fat oxidation; **(C)** forest plot of subgroup carbohydrate oxidation; **(D)** forest plot of subgroup fat oxidation; **(E)** forest plot of sports drink amount on CHO oxidation; **(F)** forest plot of sports drink amount on fat oxidation.

All five studies (Clarke et al., 2005; Hodgson et al., 2013b; Roberts et al., 2014; Garnacho-Castano et al., 2018; Pettersson et al., 2019) report the effect of the sports drink on CHO-O. The meta-analysis shows that sports drinks have no impact on the CHO-O of athletes (WMD = 0.29; 95% CI, −0.06 to 0.65, *P* = 0.106). Heterogeneity is observed (I^2^ = 97.4%, *P* < 0.001) (Figure 2A).

### Sports Drinks Have No Significant Effect on Fat-O

All five studies (Clarke et al., 2005; Hodgson et al., 2013b; Roberts et al., 2014; Garnacho-Castano et al., 2018; Pettersson et al., 2019) report the effect of the sports drink on Fat-O. The analysis of the combined trials shows that sports drinks have no impact on the Fat-O of athletes (WMD = −0.074; 95% CI, −0.19 to 0.06, *P* = 0.297). Heterogeneity is observed (I^2^ = 97.5%, *P* < 0.001) (Figure 2B).

### Carbohydrate–Electrolyte Solutions Have a Significant Effect on CHO-O

In the three studies (Clarke et al., 2005; Roberts et al., 2014; Pettersson et al., 2019) on carbohydrate–electrolyte solutions, the sports drinks increase CHO-O (WMD = 0.47; 95% CI, 0.08–0.87, *P* = 0.020). Heterogeneity is observed (I^2^ = 97.8%, *P* < 0.001). Caffeine (Hodgson et al., 2013b) (WMD = −0.02; 95% CI, −0.14 to 0.10, *P* = 0.752) and beetroot juice (Garnacho-Castano et al., 2018) (WMD = −0.26; 95% CI, −1.20 to 0.68, *P* = 0.106) have no impact on CHO-O (Figure 2C and Supplementary Table 2), but a meta-analysis could not be performed because only one trial reported about each sports drink.

### Carbohydrate–Electrolyte Solutions Have No Significant Effect on Fat-O

Carbohydrate–electrolyte solutions (Clarke et al., 2005; Roberts et al., 2014; Pettersson et al., 2019) (WMD = −0.14; 95% CI, −0.31 to 0.03, *P* = 0.103; I^2^ = 98.2%, *P* < 0.001) and beetroot juice (Garnacho-Castano et al., 2018) (WMD = 0.05; 95% CI, −0.01 to 0.11, *P* = 0.121) have no significant impact on Fat-O, while caffeine (Hodgson et al., 2013b) has a borderline effect on Fat-O (WMD = 0.05; 95% CI, 0.00–0.10, *P* = 0.050) (Figure 2D and Supplementary Table 2), but a meta-analysis could not be performed for caffeine and beetroot juice because only one trial reported about each sports drink.

### Small Intake of Sports Drinks Has a Significant Effect on CHO-O

An intake of >300 ml of sports drink (Clarke et al., 2005; Hodgson et al., 2013b) had no impact on CHO-O (WMD = 0.11; 95% CI, −0.14 to 0.37, *P* = 0.378; I^2^ = 91.7%, *P* = 0.001), but a smaller intake (<300 ml) (Roberts et al., 2014; Garnacho-Castano et al., 2018; Pettersson et al., 2019) did have an impact on CHO-O (WMD = 0.50; 95% CI, 0.12–0.87, *P* = 0.009; I^2^ = 82.6%, *P* = 0.003) (Figure 2E and Supplementary Table 2). A larger intake (WMD = 0.02; 95% CI, −0.04 to 0.17, *P* = 0.584; I^2^ = 76.2%, *P* = 0.041) or a smaller intake (WMD = −0.12; 95% CI, −0.30 to 0.06, *P* = 0.175; I^2^ = 97.1%, *P* < 0.001) had no impact Fat-O (Figure 2F and Supplementary Table 2).

### Subgroup Analyses

Supplementary Table 2 shows that the sports drinks have benefits on CHO-O in soccer (WMD = 0.24; 95% CI, 0.16–0.32, *P* < 0.001) and skiing (WMD = 0.41; 95% CI, 0.17–0.65, *P* = 0.001) but not in cycling (WMD = 0.23; 95% CI, −0.44 to 0.90, *P* = 0.501). Regarding Fat-O, sports drinks have an important benefit in skiing (WMD = −0.16; 95% CI, −0.19 to 0.06, *P* < 0.001) but not in soccer (WMD = −0.01; 95% CI, −0.04 to 0.02, *P* = 0.488) or cycling (WMD = −0.05; 95% CI, −0.27 to 0.17, *P* = 0.637).

### Sensitivity Analyses

**Figure 3:**
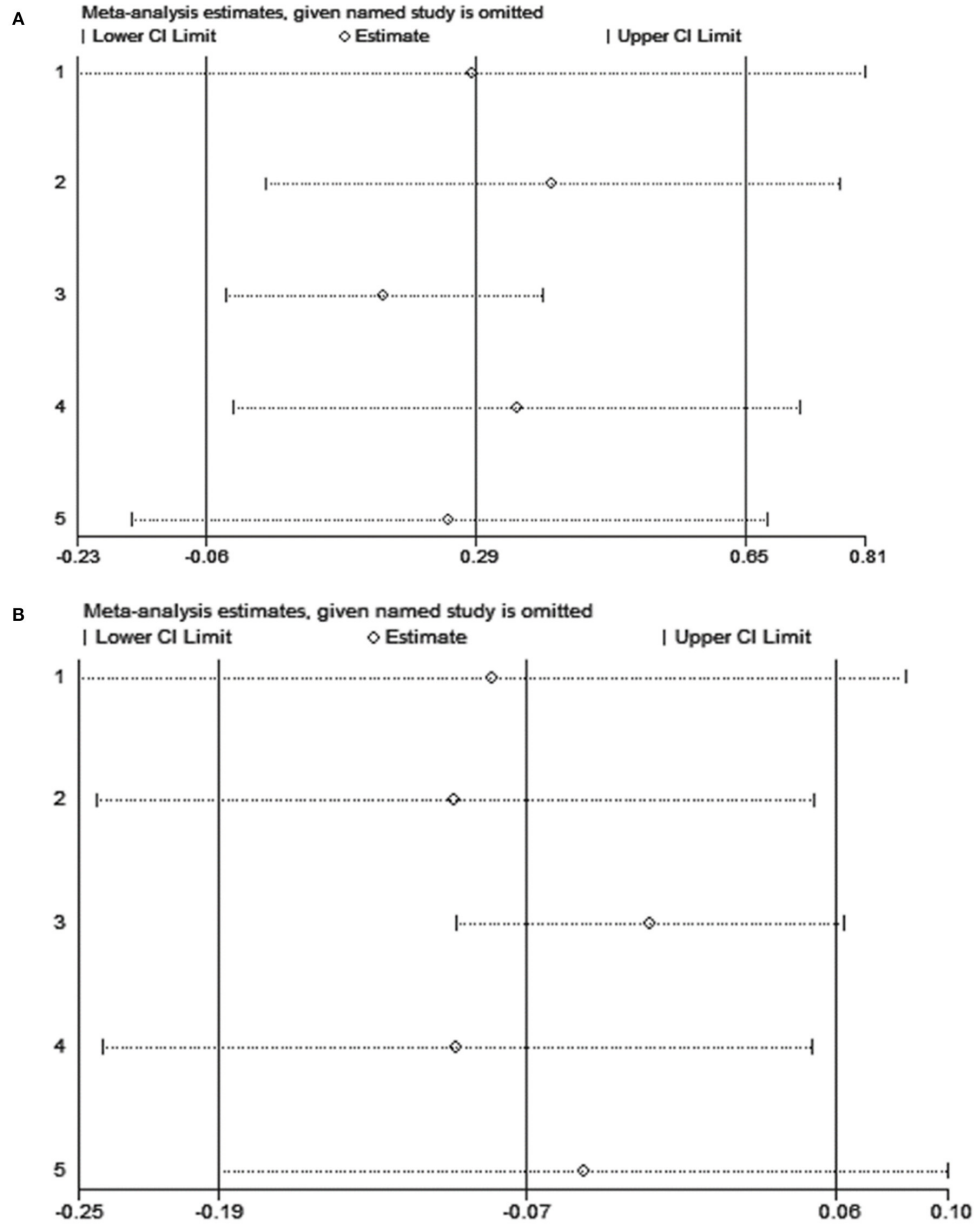
**(A)** Sensitivity analysis for CHO-O; **(B)** Sensitivity analysis for Fat-O.

Figure 3 presents the sensitivity analyses for CHO-O and Fat-O, respectively. When omitting each study sequentially, the observed effects on CHO-O and Fat-O remained consistent.

## Discussion

There is a public interest in the accuracy of the claims of commercially available sports drinks about their benefits for substrate oxidation among athletes. Therefore, the aim of this meta-analysis is to examine the effects of sports drinks ingestion during high-intensity exercise for the CHO-O among athletes. The results indicate that, compared with a placebo, sports drinks show no significant improvements on CHO-O and Fat-O in athletes. Carbohydrate–electrolyte solutions increase CHO-O in athletes but not Fat-O.

It was suggested that carbohydrate–electrolyte drinks might improve athletic performances over consecutive running sessions in men (Davison et al., 2008) and hydration after exercise-induced dehydration during running in men (Wong and Chen, 2011). On the other hand, carbohydrate–protein drinks were suggested to improve time to exhaustion during cycling in men (Saunders et al., 2004, 2007), to attenuate creatine kinase increase during cycling in men (Skillen et al., 2008), and to increase glycogen stores after cycling in men (Ivy et al., 2002; Berardi et al., 2006). A previous meta-analysis showed that the consumption of chocolate milk (which contains carbohydrates, proteins, and electrolytes) by trained athletes has no impact on the time to exhaustion, perceived exhaustion, heart rate, and serum levels of lactate and creatinine kinase (Amiri et al., 2019). On the other hand, two energy drinks containing carbohydrates and caffeine lead to superior VO_2_max and time to exhaustion compared with placebo, without effect on heart rate and blood lactate during running in men (Rahnama et al., 2010). Brink-Elfegoun et al. (2014) showed that male tennis players consuming carbohydrate–electrolyte sports drinks have no decline in physical performance over consecutive matches. Byars et al. (2010) examined the effects of a modified pre-exercise sports drink in men and women performing aerobic fitness and showed that the drink improves VO_2_max, time to exhaustion, and the estimated proportion of nonprotein fat substrate utilization, compared with placebo. The aim of this meta-analysis is to examine the effects of sports drink ingestion during high-intensity exercise on CHO-O and Fat-O, two indicators of energy consumption among athletes. The results indicate that, compared with a placebo, sports drink showed no significant improvement on CHO-O and Fat-O in athletes. The other studies described above, even though they did report performance benefits, do not report those two quantitative outcomes and are not included in the present meta-analysis. Therefore, the present meta-analysis cannot conclude that the energy drinks have no effect on sports performance but that they have no effect on metabolic indexes of energy production. Nevertheless, the present meta-analysis and the above studies seem to conflict in terms of conclusions because of the different outcome measures, i.e., quantitative vs. semiquantitative or qualitative. The sports drinks included in the meta-analysis could still have effects on other sports outcomes such as exhaustion perception, hydration, and recuperation, but those outcomes are not examined in the present meta-analysis. A meta-analysis revealed that carbohydrate drinks improve sports endurance (Vandenbogaerde and Hopkins, 2011), but it does not examine CHO-O. Future studies about sports drinks should be carefully designed by including quantitative metabolic indexes that could explain the semiquantitative/qualitative indexes of sports performance.

When considering each of the five studies included in this meta-analysis, three studies (Clarke et al., 2005; Roberts et al., 2014; Pettersson et al., 2019) actually show higher CHO-O with the sports drinks (in male soccer players, male cyclists, and male cross-country skiers, respectively), while two studies (Hodgson et al., 2013b; Garnacho-Castano et al., 2018) (in male cyclists/triathletes and male triathletes, respectively) show no effect. Nevertheless, the sensitivity analysis shows that the sequential exclusion of any one of those study significantly did not change the result of this meta-analysis. Similarly, two studies (Roberts et al., 2014; Pettersson et al., 2019) show a decrease in Fat-O with sports drink, one study (Hodgson et al., 2013b) report an increase, while two studies (Clarke et al., 2005; Garnacho-Castano et al., 2018) show no effect. Again, the exclusion of any one study does not change the conclusion.

The present study includes three types of energy drinks: carbohydrate–electrolyte, caffeine, and beetroot juice (mainly carbohydrates). The analyses of all three types together show that energy drinks have no impact on CHO-O and Fat-O, but the subgroup analyses show that carbohydrate–electrolyte drinks have a positive impact on CHO-O, while the caffeine drink decreases Fat-O. This is in agreement with the view that carbohydrate–electrolyte drinks improve repeated exercise performance in men after running (Davison et al., 2008; Kalman et al., 2012) and workout (Orru et al., 2018). A study also reported high CHO-O with the use of carbohydrate–electrolyte gels in male cyclists (Willems et al., 2011). The higher rate of CHO-O observed with carbohydrate–electrolyte consumption might be due to readily available source of energy that allows the preservation of glycogen stores, decreases fatigue perception, and prevents acute hypoglycemia (Coggan and Coyle, 1987; Bosch et al., 1994; Jeukendrup, 2004; Willems et al., 2011; King et al., 2018), with positive effects on time trials (Currell and Jeukendrup, 2008; Triplett et al., 2010) and power output (Currell and Jeukendrup, 2008; Rowlands et al., 2012). In addition, the use of transportable carbohydrates also increases the transport of water from the intestine, improving hydration (Jentjens et al., 2006; Jeukendrup et al., 2009; Jeukendrup, 2010; Jeukendrup and Moseley, 2010). Regarding caffeine, green tea extract can increase Fat-O (Hodgson et al., 2013a). Caffeine increases Fat-O in hepatocytes (Sinha et al., 2014), and caffeine increases exercise tolerance (Kumar et al., 2019). Therefore, future studies should examine individual types of energy drinks because of their different metabolic effects.

The results of this meta-analysis must be considered together with its limitations. First, the outcomes of interest might be biased by the studies we included since they were conducted at various institutions in different countries. The baseline characteristics of athletes from different studies and experimental trials in each study are different, as well. Second, the outcome parameters are estimated from the provided figures if the parameters are not reported in the original documents, probably introducing biases. Third, the amounts of beverage ingested are different in different studies. Fourth, all included studies are crossover studies. Even though the effect between two consecutive ingestion of sports drinks might be nonsignificant, there is a risk of carry-out bias. Finally, due to the population in each study being elite athletes, and the experimental trial included high-endurance exercise, the applicability of the results should be limited to such a population. In addition, the total number of participants in each study is small. Only 58 participants are included in our meta-analysis.

## Conclusion

In conclusion, compared with placebo, sports drinks show no significant improvement on CHO-O and Fat-O in athletes. Carbohydrate–electrolyte solutions increase CHO-O in athletes but not Fat-O. Randomized controlled trials with large sample sizes should be conducted to investigate the effectiveness of sports drinks for athletes during exercise.

## Data Availability Statement

The original contributions presented in the study are included in the article/Supplementary Material, further inquiries can be directed to the corresponding author/s.

## Author Contributions

XL and CW carried out the studies, participated in collecting data, and drafted the manuscript. AW, WW, and RG participated in the acquisition, analysis, or interpretation of data and drafted the manuscript. All authors contributed to the article and approved the submitted version.

## Conflict of Interest

The authors declare that the research was conducted in the absence of any commercial or financial relationships that could be construed as a potential conflict of interest.
