# Supplementary material for: The Effects of Sports Drinks During High-Intensity Exercise on the Carbohydrate Oxidation Rate Among Athletes: A Systematic Review and Meta-Analysis
Source: Front Physiol. 2020 Dec 11;11:574172. doi: 10.3389/fphys.2020.574172 (PMC7759561; doi:10.3389/fphys.2020.574172)
Supplement: Supplementary file 1 [file Data_Sheet_1.PDF]

Supplementary Table S1. Cochrane criteria for quality of RCT.

Study	Random sequence generation (selection bias)	Allocation concealment (selection bias)	Blinding of participants and personnel (performance bias)	Blinding of outcome assessment (detection bias)	Incomplete outcome data (attrition bias)	Selective reporting (reporting bias)	Other bias
Clarke, 2005	High	High	Low	Low	Low	Low	Low
Hodgson, 2013	High	Low	High	Low	Low	Low	Low
Robert, 2014	High	Low	Low	Low	Low	Low	Low
Garnacho-Castano, 2018	High	Low	Low	Low	Low	Low	Low
Petterson, 2019	High	High	Low	Low	Low	Low	Low

Supplementary Table S2. Subgroup analyses

	N	WMD (95%CI)	P	I^2	P (Heterogeneity)
CHO-O	5	0.29 (-0.06,0.65)	0.106	97.4%	<0.001
CHO-E	3	0.47 (0.08,0.87)	0.020	97.8%	<0.001
Caffeine	1	-0.02 (-0.14,0.10)	0.752	.	.
Beetroot Juice	1	-0.26 (-1.20,0.68)	0.106	.	.
Soccer	1	0.24 (0.16,0.32)	<0.001	.	.
Cycling	3	0.23 (-0.44,0.90)	0.501	98.3%	<0.001
Ski	1	0.41 (0.17,0.65)	0.001	.	.
>300 ml	2	0.11(-0.14,0.37)	0.378	91.7	0.001
<300 ml	3	0.50(0.12,0.87)	0.009	82.6	0.003
Fat-O	5	-0.07 (-0.19,0.06)	0.297	97.5%	<0.001
CHO-E	3	-0.14 (-0.32,0.03)	0.103	98.2%	<0.001
Caffeine	1	0.05 (0.00,0.10)	0.050	.	.
Beetroot Juice	1	0.05 (-0.01,0.11)	0.121	.	.
Soccer	1	-0.01 (-0.04,0.02)	0.488	.	.

Cycling	3	-0.05 (-0.28,0.17)	0.637	98.4%	<0.001
Ski	1	-0.16 (-0.19,0.06)	<0.001	.	.
>300 ml	2	0.02(-0.04,0.07))	0.584	76.2	0.041
<300 ml	3	-0.12(-0.30,0.06)	0.175	97.1	<0.001

WMD: weighted mean difference; CI: confidence interval; CHO-O: carbohydrate oxidation rate; CHO-E: carbohydrate-electrolyte drink; Fat-O: fat oxidation rate.
